# Increased metabolic activity in the septum and habenula during stress is linked to subsequent expression of learned helplessness behavior

**DOI:** 10.3389/fnhum.2014.00029

**Published:** 2014-02-03

**Authors:** Martine M. Mirrione, Daniela Schulz, Kyle A. B. Lapidus, Samuel Zhang, Wayne Goodman, Fritz A. Henn

**Affiliations:** ^1^Biomedical Sciences Department, Quinnipiac UniversityHamden, CT, USA; ^2^Cold Spring Harbor Laboratory, NeuroscienceCold Spring Harbor, NY, USA; ^3^Brookhaven National Laboratory, Medical DepartmentUpton, NY, USA; ^4^Department of Neurobiology and Behavior, Stony Brook UniversityStony Brook, NY, USA; ^5^Psychiatry Department, Icahn School of MedicineNew York, NY, USA

**Keywords:** metabolism, whole-brain, imaging, FDG, PET, circuitry, depression, DBS

## Abstract

Uncontrollable stress can have a profound effect on an organism's ability to respond effectively to future stressful situations. Behavior subsequent to uncontrollable stress can vary greatly between individuals, falling on a spectrum between healthy resilience and maladaptive learned helplessness. It is unclear whether dysfunctional brain activity during uncontrollable stress is associated with vulnerability to learned helplessness; therefore, we measured metabolic activity during uncontrollable stress that correlated with ensuing inability to escape future stressors. We took advantage of small animal positron emission tomography (PET) and 2-deoxy-2[^18^F]fluoro-D-glucose (^18^FDG) to probe *in vivo* metabolic activity in wild type Sprague Dawley rats during uncontrollable, inescapable, unpredictable foot-shock stress, and subsequently tested the animals response to controllable, escapable, predictable foot-shock stress. When we correlated metabolic activity during the uncontrollable stress with consequent behavioral outcomes, we found that the degree to which animals failed to escape the foot-shock correlated with increased metabolic activity in the lateral septum and habenula. When used a seed region, metabolic activity in the habenula correlated with activity in the lateral septum, hypothalamus, medial thalamus, mammillary nuclei, ventral tegmental area, central gray, interpeduncular nuclei, periaqueductal gray, dorsal raphe, and rostromedial tegmental nucleus, caudal linear raphe, and subiculum transition area. Furthermore, the lateral septum correlated with metabolic activity in the preoptic area, medial thalamus, habenula, interpeduncular nuclei, periaqueductal gray, dorsal raphe, and caudal linear raphe. Together, our data suggest a group of brain regions involved in sensitivity to uncontrollable stress involving the lateral septum and habenula.

## Introduction

Major depression affects 16.6% of the adult U.S. population (lifetime prevalence) and less than 20% of patients afflicted are receiving adequate treatment (Kessler et al., 2005a,b; Wang et al., 2005). The learned helplessness paradigm is a well-established animal model of depressive- and anxiety-like behavior (Overmier and Seligman, 1967) with good predictive and face validity (Vollmayr and Henn, 2003), and potentially useful for identifying new targetable pathways for the treatment of depression (Li et al., 2011). Learned helplessness is based on the concept that some individuals perceiving their environment as out of their control and unmanageable lose the ability to cope with future stressors, which can result in anxiety and depressive symptoms (Maier, 1984; Forgeard et al., 2011). In rats, the unmanageable condition is induced by a brief session of uncontrollable, unpredictable, inescapable foot-shock, and behavior is subsequently evaluated when the animal is now given control to shut off the predictable foot-shocks with a lever press. The degree to which animals control their environment by terminating the foot-shock classifies them on a spectrum from resilience to helplessness. The mechanisms driving helplessness behavior are complex, but include deficits in reinforcement learning, working memory, and abnormalities in monoamines (Schulz et al., 2010). Notably, wild type animals show individual differences in degree of helplessness when exposed to this paradigm similar to the varying susceptibility to stress seen in human major depression.

Metabolic brain activity associated with major depression has been explored with ^18^FDG-PET neuroimaging revealing complex changes in the prefrontal cortex, thalamus, limbic system, and basal ganglia of depressed patients (Price and Drevets, 2012). Previous studies have shown analogous changes in metabolic activity associated with depressive-like behavior in animal models, using [C14]2-deoxyglucose (2-DG) (Caldecott-Hazard et al., 1988) and cytochrome oxidase staining (Shumake et al., 2000, 2001, 2002, 2003), demonstrating similar pathological end-points compared to human patients. Additionally, ^18^FDG-PET measurements during immobilization stress have shown decreased metabolic activity in the hippocampus associated with increased hypothalamic activity (Sung et al., 2009), and the reduced hippocampal activity observed on the first day of a forced swim test was resolved with fluoxetine administration (Jang et al., 2009). However, it is unclear what factors contribute to terminal metabolic pathology in depressed patients and in models such as learned helplessness. As an *in vivo* measurement, ^18^FDG-PET is particularly amenable to examining neural activity over the time course of a disease, thus allowing us to identify brain activity related the development of learned helplessness. We hypothesized that the source of metabolic dysfunction could be traced to metabolic signatures associated with initial stressful events, prior to expression of depressive-like behavior. Therefore, we used ^18^FDG-PET to measure metabolic activity related to uncontrollable stress that subsequently correlated with degree of learned helplessness expression.

## Materials and methods

### Animals

Male Sprague-Dawley rats (*n* = 12) were purchased from Taconic Farms and allowed to acclimate to the animal facility for 2 weeks prior to experiments. The rats were housed 2 per cage under a 12–12 h light-dark cycle (7 am–7 pm) with food and water freely available. The animals were between 3–5 months old and weighed 350–500 g at the time of behavioral imaging. All procedures involving animals were approved by Brookhaven National Laboratory Institutional Animal Care and Use Committee and conducted at Brookhaven National Laboratory.

### Learned helplessness paradigm

Operant chambers (Coulbourn Instruments, PA) were 30.5 × 24.5 × 30.5 cm and the experiments were conducted between 0900 and 1100 to minimize the effect of circadian rhythm. Methods for the learned helplessness paradigm have been optimized previously (Vollmayr and Henn, 2001; Schulz et al., 2010). In brief, animals were exposed to 120 uncontrollable, inescapable foot-shocks at 0.4 mA over 40 min, with unpredictable shock duration and inter-shock intervals (ITI's) ranging from 5 to 15 s. Typically on the following day (but for this experiment forty 8 h later to accommodate radiotracer decay), an illuminated lever that controls shock termination was added to the chamber for escape testing. Rats had the opportunity to press the lever to escape a maximum of 15 trials comprised of foot-shocks lasting up to 60 s (shorter if terminated by a lever press), and with predictable ITI's of 24 s. For increased stringency, only lever presses occurring within the first 20 s of shock onset were counted.

### Small animal pet imaging

Directly following uncontrollable inescapable foot-shock, animals were transported to the PET facility and injected with ^18^FDG (~2 mCi/kg, intraperitoneal injection, manufactured by Cardinal Health or Brookhaven National Laboratory cyclotron) in the home cage with a 45 min uptake period allowing the radiotracer to become trapped in metabolically active cells (Gallagher et al., 1978). In order to maximize radiotracer uptake, all animals were food deprived for a total of 3–4 h prior to radiotracer administration. Rats were anesthetized (100 mg/kg Ketamine/Xylazine), and underwent a 10 min static acquisition scan in a microPETR4 tomograph (Concord Microsystems, Knoxville, TN), which has a spatial resolution of 1.85 mm full width at half maximum (FWHM) in the axial direction, and 1.66 mm FWHM in the transaxial direction at the center of the field of view (FOV) (Knoess et al., 2003).

Following the scan, a venous blood sample was collected from the tail of each subject for measuring glucose (CVS brand glucometer), which was determined to be within the normal glycemic range (Toyama et al., 2004) for each subject (11.26 ± 2.11 mmol/L, overall mean ± standard deviation). Sinograms were corrected for photon scatter (Alexoff et al., 2003) and were reconstructed using the Ordered Subsets Expectation-Maximization 3-dimensional/ maximum *a posteriori* (OSEM3D/MAP) algorithm with 2 OSEM3D and 18 MAP iterations, and pixel size of 0.8 × 0.8 × 1.2 mm.

### Pet data analysis

Biomedical Image Quantification software PMOD version 2.9 and the Fusion Toolbox (PMOD Technologies Ltd) were used for image processing and region of interest (ROI) analysis. Statistical Parametric Mapping version 8 (SPM, Welcome Dept. of Cognitive Neurology, London, UK) run in Matlab 2010b (Math-works Inc.) was also used for spatial pre-processing (co-registration, normalization) and statistics. SPM analysis is a voxel based approach that detects statistically significant changes within the brain without a predetermined hypothesis, initially developed by Friston et al. (1991).

Following a similar strategy previously employed (Mirrione et al., 2007), we created an ^18^FDG-rat-template (voxel size 2.0 × 2.0 × 2.0 mm) for accurate alignment of subjects in this study. A group of naive subjects (*n* = 10) were co-registered and enlarged by a factor of 10 to match the coordinate space of an MRI reference template (Schweinhardt et al., 2003), averaged, and smoothed with an isotropic Gaussian kernel (6 mm FWHM) to remove any subtle non-uniformities. Spatial transformation of each subject to fit standardized rat brain coordinate space minimizes analysis variability due to slightly different brain shape, size or position.

The PET scan for each subject in the study was then aligned to the ^18^FDG-rat-template. Co-registration and normalization parameters included normalized mutual information and trilinear interpolation for ridged transformations, and affine regularization (preserved concentrations) for non-linear transformations. Skull stripping (removal of PET signal from outside of the brain) and global normalization (scaling all images to their whole brain value) were completed in PMOD. Global normalization yields a parametric image for each scan on an identical scale by normalizing for variability in injected ^18^FDG dose between subjects, yielding a reliable measure of regional metabolic activity suitable for across subject ROI comparisons.

**Figure 1:**
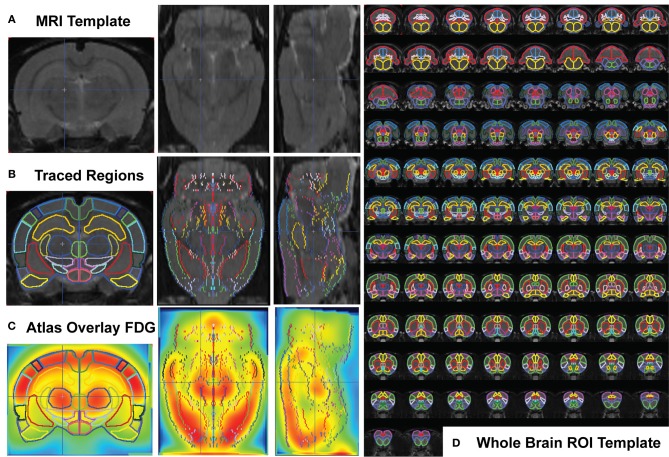
**Whole Brain PET Analysis. (A)** T2-weighted rat template (Schweinhardt et al., 2003) in coronal (left), transverse (middle), and sagittal (right) views. **(B)** Each slice of the template (left half of symmetrical coronal section) was hand traced using the PMOD Biomedical Image Quantification Software (www.pmod.com), region-of-interest (ROI) analysis tool, and each completed ROI flipped symmetrically to the right side. **(C)** The whole-brain ROI template was overlaid on each subject's whole-brain PET image (after spatial preprocessing to align each individual animal to the ^18^FDG-rat-template, shown). **(D)** Whole brain ROI template containing 57 separated brain regions, drawn over 90 coronal template sections.

A ROI template for whole-brain quantification of the ^18^FDG-PET signal was created using the MRI reference template (Schweinhardt et al., 2003) and Paxinos and Watson 6th edition Rat Brain Atlas (Paxinos and Watson, 2007) (Figures 1A–C). Fifty-seven brain regions (+4.8 through −13.0 mm to bregma) were designated with many functionally similar small regions combined into single ROIs, conforming to the spatial resolution range for small animal PET. ^18^FDG uptake was calculated in each ROI of each subject using the individual normalized parametric images and the in-house generated ROI-template (Figure 1D, overlaid on MRI -template). A labeled map of the ROI-template is provided (Supplemental Figure 1). Readers interested in using the in-house generated rat whole-brain ROI template for data analysis may contact the corresponding author.

### Statistics

Pearson product-moment correlation coefficients “*r*”) were measured using GraphPad Prism Version 4 with a statistical threshold of *p* < 0.05. Test completion time or number of successful lever presses terminating the shock were used as behavioral parameters and correlated with regional ^18^FDG uptake. Normalized parametric images were also evaluated with SPM to pictorially display the distribution of voxels that correlated with behavior. Time to finish the test was used as a covariate in a one-sample *t*-test (uncorrected, *p* < 0.05).

## Results

All animals received an identical pattern and duration of uncontrollable foot-shock exposure directly followed by ^18^FDG metabolic measurements. Forty-eight hours later, animals were tested for propensity to escape foot-shock stress. The distribution of escape behavior is listed in Table 1. Since pressing the lever terminates the shock and immediately begins the next inter-trial interval (and escape trial), rats with fewer lever presses take longer to finish the test. On the behavioral spectrum, animals that do not press the lever at all (taking the maximum time to finish the test) are considered the most helpless, whereas animals that press the lever for every trial (maximum of 15) and taking less time to finish the test are considered the most resilient.

**Table 1:**
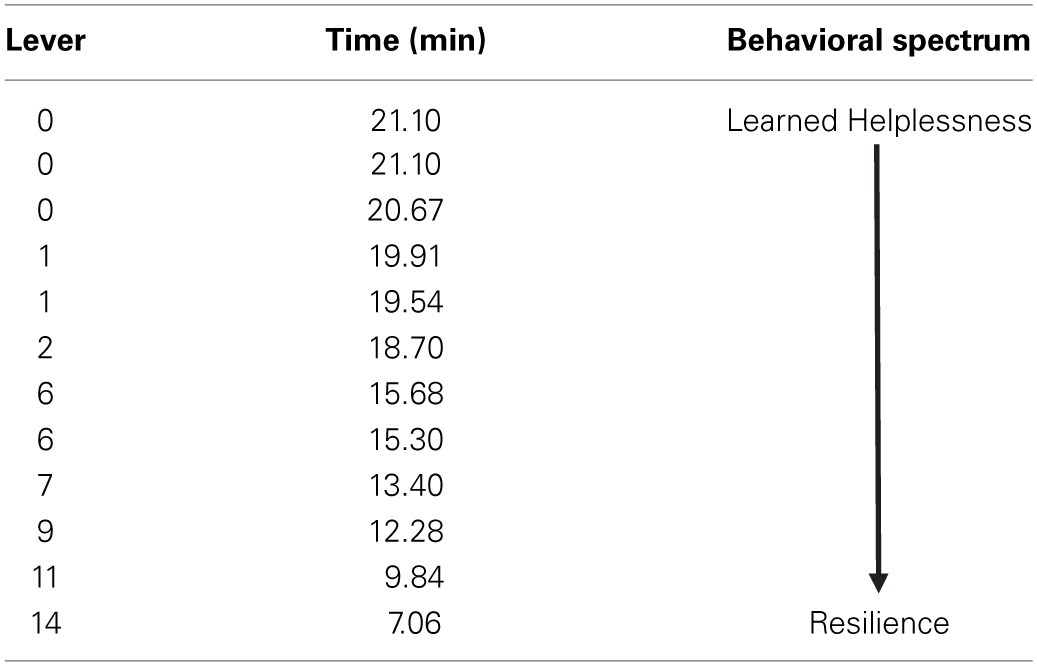
**Distribution of escape behavior**.

**Figure 2:**
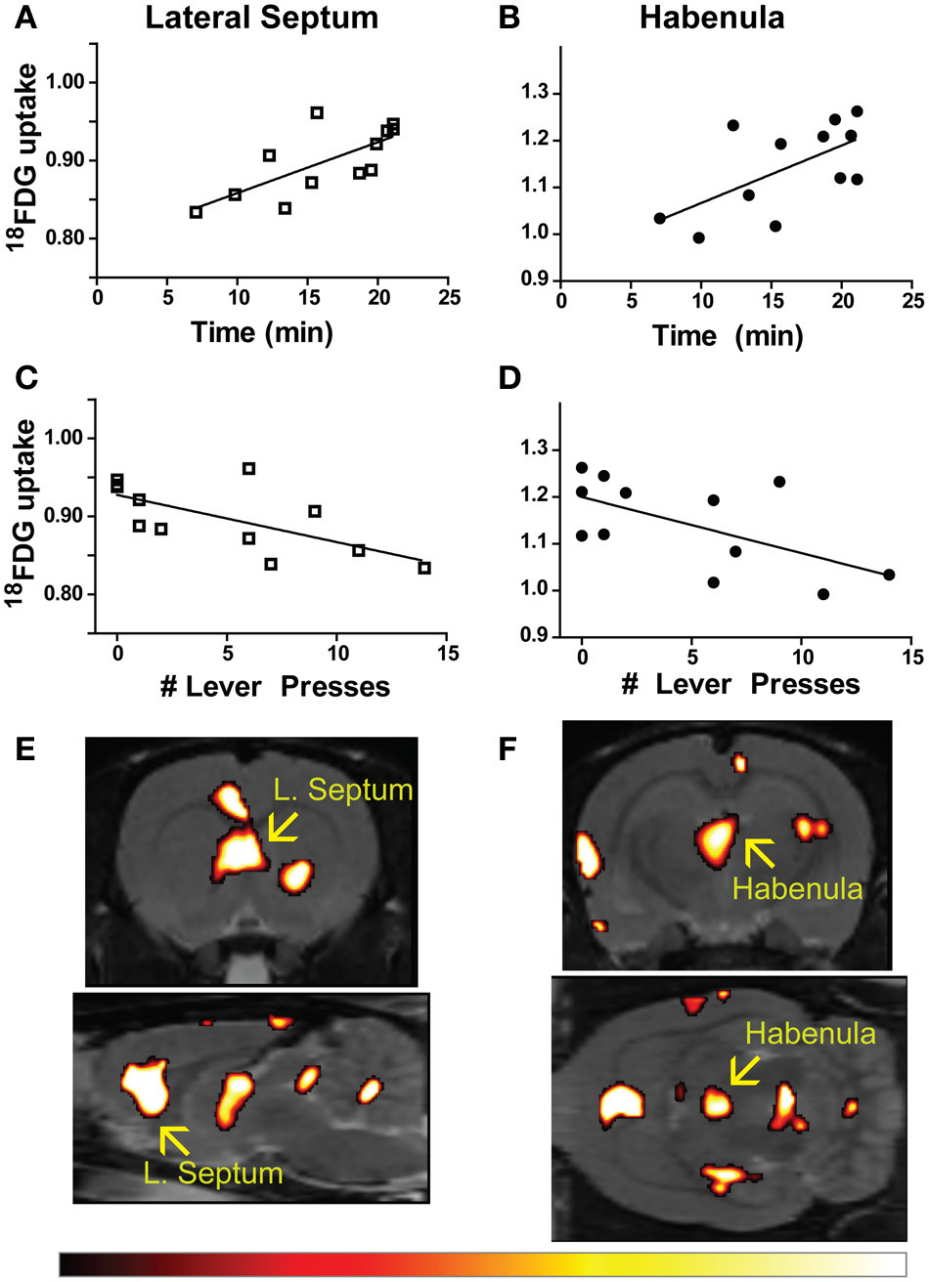
**Brain-behavior correlations in learned helplessness. (A–D)** Time to finish the test **(A,B)** and number of lever presses within 20 s of shock onset **(C,D)** correlated with metabolic activity in the lateral septum (time: *r* = 0.7131, *p* = 0.0092; lever: *r* = −0.6692, *p* = 0.0173), and habenula (time: *r* = 0.6138, *p* = 0.0338; lever: *r* = −0.6060, *p* = 0.0367). **(E,F)** Metabolic activity correlating with time to finish the test visualized with a voxel-by-voxel one sample *t*-test (*p* < 0.05), arrows designate the lateral septum **(E)** and habenula **(F)** as bright areas overlaid on the T2-weighted rat template using the hot color scale (shown below).

**Figure 3:**
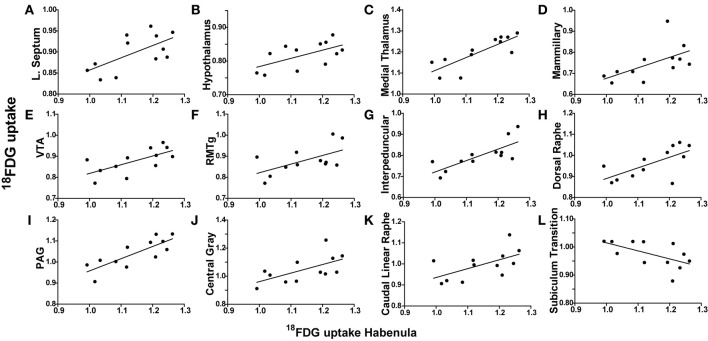
**Habenula metabolic activity regional correlates. (A–L)** ^18^FDG uptake in the habenula (x-axes) correlates with ^18^FDG uptake in several brain regions (y-axes) including the lateral septum **(A)**, hypothalamus **(B)**, medial thalamus **(C)**, mammillary nuclei **(D)**, ventral tegmental area (VTA) **(E)**, rostromedial tegmental nucleus (RMTg) **(F)**, interpeduncular nuclei **(G)**, dorsal raphe **(H)**, periaqueductal gray (PAG) **(I)**, central gray **(J)**, caudal linear raphe **(K)**, and subiculum transition area **(L)**.

Time to finish the test and lever press number were correlated with metabolic activity in 57 individual regions-of-interest using the in-house generated rat-ROI-template. Pearson product-moment correlation coefficients (“*r*”) were generated using time (min) or number of lever presses on the x-axis, and averaged bilateral metabolic activity for each brain region on the y-axis. Correlations (uncorrected *p* < 0.05, two-tailed) were discovered for both time (Figures 2A,B) and lever (Figures 2C,D) in the lateral septum (time: *r* = 0.7131, *p* = 0.0092; lever: *r* = −0.6692, *p* = 0.0173) and habenula (time: *r* = 0.6138, *p* = 0.0338; lever: *r* = −0.6060, *p* = 0.0367). A voxel-by-voxel one sample *t*-test using time required to finish the test as a covariate was generated (uncorrected *p* < 0.05) to visualize the positive correlations between enhanced activity in the lateral septum or habenula with escape deficits (Figures 2E,F arrows demarcate regions that corresponded to the whole-brain ROI analysis). Other brain areas visualized in Figures 2E,F did not reach threshold for reporting using the ROI analysis. The SPM analysis highlighted that activity in the anterior portion of the lateral septum (in the same coronal plane as the nucleus accumbens) correlated with behavior, whereas the medial septum or posterior septum ROIs (as segmented it in our template, see Supplemental Figure 1 for anatomical locations) did not reach threshold for reporting (ROI analysis medial septum: *r* = 0.3610 and *p* = 0.2490; ROI analysis posterior septum: *r* = −0.03424 *p* = 0.9159; data not shown). For both the lateral septum and habenula, metabolic activity during uncontrollable stress positively correlated with increased time to finish the test and negatively correlated with number of lever presses. Furthermore, metabolic activity in the lateral septum and habenula were positively correlated with each other (*r* = 0.6390, *p* = 0.0253) (Figure 3A). The six animals that had the fewest lever presses (≤2) are represented by the points closest to each other in Figures 2A–D and by the elevated metabolic activity in the habenula and lateral septum (Figure 3A).

To determine whether metabolic activity in additional brain regions varied in association with the lateral septum or habenula, Pearson product-moment correlation coefficients (“*r*”) were generated for comparison with the remaining 56 ROIs. When the habenula was used as a seed region, its metabolic activity was positively correlated with the hypothalamus (*r* = 0.5942, *p* = 0.0416), medial thalamus (*r* = 0.8153, *p* = 0.0012), mammillary nuclei (*r* = 0.5835, *p* = 0.0464), ventral tegmental area (VTA) (*r* = 0.6654, *p* = 0.0182), rostromedial tegmental nucleus (RMTg) (*r* = 0.5876, *p* = 0.0445), interpeduncular nuclei (*r* = 0.7695, *p* = 0.0034), dorsal raphe (*r* = 0.6702, *p* = 0.0171), periaqueductal gray (PAG) (*r* = 0.8020, *p* = 0.0017), central gray (*r* = 0.6108, *p* = 0.0349), and caudal linear raphe (*r* = 0.5917, *p* = 0.0427), and negatively correlated with the subiculum transition area (*r* = −0.5903, *p* = 0.0433). Individual correlation plots for region pairs are shown for the habenula (Figures 3B–L). These data reveal that individual animals with elevated habenula activity had correspondingly similar increases (or decrease) in activity levels of additional brain regions.

**Figure 4:**
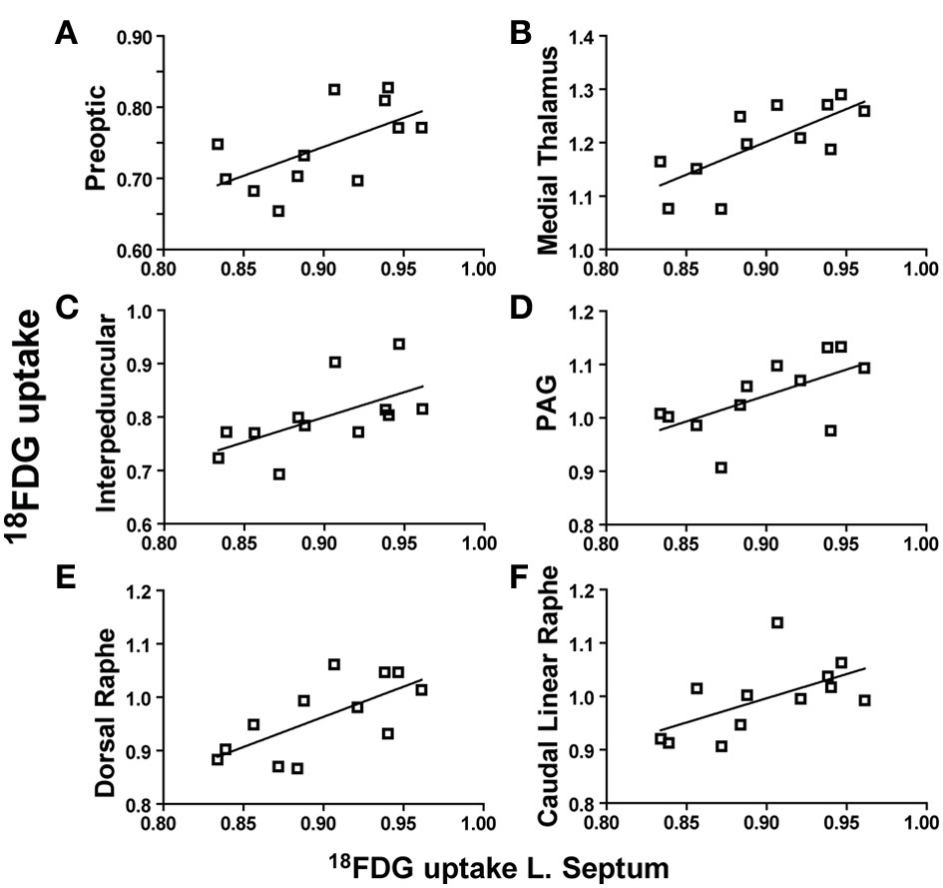
**Lateral septum metabolic activity regional correlates. (A–F)** ^18^FDG uptake in the lateral septum (x-axes) correlates with ^18^FDG uptake in several brain regions (y-axes) including the preoptic area **(A)**, medial thalamus **(B)**, interpeduncular nuclei **(C)**, periaqueductal gray (PAG) **(D)**, dorsal raphe **(E)**, and caudal linear raphe **(F)**.

When the lateral septum was used as a seed region, its metabolic activity was positively correlated with the preoptic area (*r* = 0.6066, *p* = 0.0365), medial thalamus (*r* = 0.7314, *p* = 0.0069), interpeduncular nuclei (*r* = 0.6040, *p* = 0.0367), PAG (*r* = 0.6134, *p* = 0.0339), dorsal raphe (*r* = 0.6835, *p* = 0.0143), and the caudal linear raphe (*r* = 0.5821, *p* = 0.0471). Individual correlation plots for region pairs are shown for the lateral septum (Figures 4A–F). These data reveal that individual animals with elevated lateral septum activity had correspondingly similar increases in activity levels of additional brain regions.

We analyzed the habenula and lateral septum as seed regions given their correlation with behavior. However, further exploration of the whole brain data set through a region-by-region correlation analysis, without an *a priori* hypothesis of particular regions to use as seeds, could also be conducted to reveal additional relationships between regional activity levels. A region that did not correlate with behavior, the dorsal hippocampus, was used as an example seed region. The dorsal hippocampus correlated with the olfactory cortex (*r* = 0.5804, *p* = 0.0478) and ventral hippocampus (*r* = 0.6900, *p* = 0.0130), but not with the habenula, septum or any other brain region (data not shown).

## Discussion

Using the rat learned helplessness model, we have examined *in vivo* metabolic signatures associated with the development of depressive-like behavior induced by uncontrollable stress. We have identified the lateral septum and habenula as being central in a network of brain regions whose activation during uncontrollable stress correlates with subsequent degree of helplessness behavior. Our data support and extend upon existing literature suggesting a critical role for both the lateral septum and habenula in regulating mood circuitry and coping mechanisms in response to stress, and provide additional supportive rationale for investigating the habenula as a potential therapeutic target for the treatment of depression.

The lateral septum has previously been implicated in affective regulation through its role integrating excitatory input from the hippocampus and relaying information to the hypothalamus and monoaminergic/cholinergic midbrain, as well as through its interconnections with the periaqueductal gray, medial septum, amygdala, bed nucleus of the stria terminalis, medial prefrontal cortex and entorhinal cortex (Sheehan et al., 2004). Several lines of data suggest that blunted lateral septal synaptic excitability precipitates depressive-like behavior, whereas increased synaptic excitability is associated with hippocampal dependent synaptic plasticity necessary to develop active coping strategies (Urban et al., 1995; Steciuk et al., 1999; Sheehan et al., 2004). The lateral septum contains highly interconnected GABAergic projection neurons (Zhao et al., 2013); therefore the metabolic increases we report here in animals vulnerable to developing helplessness may result from enhanced excitatory input and glutamate recycling with enhanced recurrent inhibitory collaterals leading to subsequent escape deficits.

When injected directly into the lateral septum, antidepressants can reduce depressive-like behavior through a serotonergic mediated reduction in exaggerated septal recurrent collaterals (Sheehan et al., 2004; Molina-Hernández et al., 2012). Similarly, bilateral ibotinic acid lesions of the lateral septum reduce active coping strategies in the forced swim test, whereas intra-lateral septal 5-HT_1A_ agonist infusion increases active coping (Singewald et al., 2010). Furthermore, lateral septum 5-HT_1A_ receptor function is altered in the Flinders Sensitive Line model of depressive-like behavior (Yu et al., 2003), and the serotonin metabolite 5-HIAA is elevated in the lateral septum of animals that are resilient to helplessness (Ronan et al., 2000). Our data align with and provide direct support for the hypothesis that inherent differences in lateral septal activity could underlie individual differences in stress coping behaviors. The lateral septum modulates the medial septum and thus may also indirectly modulate the habenula (Yamaguchi et al., 2013), and as discussed below, habenular hyperactivity and dysfunction in dorsal raphe serotonergic innervation may feed into altered activity in the lateral septum.

The habenula acts as a relay station between forebrain (limbic and basal ganglia) and midbrain structures through complex interconnections that modulate monoaminergic output (Lecourtier and Kelly, 2007; Bianco and Wilson, 2009). Habenula activation has been shown to be associated with failure to receive an expected reward (“disappointment”) and anticipation of an aversive outcome in primates (Matsumoto and Hikosaka, 2007, 2009, 2011; Bromberg-Martin et al., 2010), and in humans (Ullsperger and Von Cramon, 2003; Salas et al., 2010), acting in contrast to dopaminergic neurons. A collaborative study from our laboratory has shown that habenula neurons in helpless animals receive increased excitatory input compared to controls on neurons projecting to the ventral tegmental area, and the degree of synaptic potentiation positively correlated with the animal's helplessness behavior (Li et al., 2011). Interestingly, expression of the beta form of calcium/calmodulin-dependent protein kinase type II was shown to be significantly up regulated in the lateral habenula of helpless animals suggesting a molecular mediator of enhanced synaptic potentiation (Li et al., 2013). When a major source of input to the habenula from the basal ganglia is specifically activated, animals display avoidance behavior consistent with the notion that habenula activation is aversive (Shabel et al., 2012). As well, direct optogenetic activation of lateral habenula efferents onto the dopaminergic midbrain have also been shown to be aversive specifically through neurons that synapse onto GABAergic neurons in the rostromedial tegmental nucleus (Stamatakis and Stuber, 2012) and medial prefrontal cortical projecting dopaminergic neurons in the ventral tegmental area (Lammel et al., 2012). Overall, modulatory efferents from the habenula to the major monoaminergic nuclei (dorsal raphe, ventral tegmental area, and locus coeruleus) are consistent with these findings and the monoamine hypothesis of depression.

Several lines of evidence suggest that behavioral helplessness resulting from uncontrollable stress is caused by impaired medial prefrontal cortical top-down inhibitory control over stress responsive limbic and habenula activated brainstem structures (Amat et al., 2005; Maier and Watkins, 2010). The habenula has been shown to drive serotonin release in the dorsal raphe during uncontrollable stress, which is necessary for expression of behavioral helplessness (Maier et al., 1995; Grahn et al., 1999; Amat et al., 2001). In humans, tryptophan depletion has been found to induce increased habenula and dorsal raphe activity, which has been associated with negative mood (Morris et al., 1999; Roiser et al., 2009). Recently, habenula hyperactivity has been reported in severe depression and decreases have been noted following antidepressant treatment (Nugent et al., 2013). These data, coupled with rodent studies demonstrating habenula-stimulation induced impairments in sucrose preference and lesion-induced increases in hedonic activity (Friedman et al., 2011) along with elevations in habenula activity in congenital learned-helplessness strain (Shumake et al., 2003), support exploration of habenula circuitry as a potential target in treating depression.

We have previously shown that reducing hyperactivity in the lateral habenula with high frequency stimulation, mimicking clinical deep brain stimulation (DBS), reduced helplessness and prevented the increase in immobility typically associated with forced swim test exposure (Li et al., 2011). DBS to this region also improved depression-like symptoms following chronic stress (Meng et al., 2011), providing support for the hypothesis that habenula DBS could be effective in alleviating intractable depression in humans (Sartorius and Henn, 2007). A severe and treatment-resistant case was selected for a trial of habenula DBS and a gradual response was observed (Sartorius et al., 2010). The patient's course was remarkable for recurrence of depressive symptoms on several occasions when the DBS was inadvertently discontinued. Taken together, the evidence suggests that the lateral habenula is overactive in depressed states, and can modulate midbrain activity contributing to depression. Therefore the aim of DBS would be to limit habenula hyperactivity and thus reduce depressive symptoms. Ongoing studies are exploring the effect of decreasing output of the habenula through DBS to ameliorate the negative effects of stress in severely depressed patients (e.g., clinicaltrials.gov identifier: NCT01798407).

We have shown correlations between uncontrollable stress and activation of lateral septum and habenula pathways coinciding with development of escape deficits and learned helpless behavior. Our data provide a potential mechanism through which vulnerability to uncontrollable stress is initiated. Further studies will evaluate whether these pathways are inherently altered in vulnerable subjects. In total, our data along with an emerging literature suggests that decreasing output of the habenula could result in increased resistance to the effects of stress and provide a rationale for inhibition of the habenula via DBS in severely depressed patients.

## Author contributions

The study was designed and analyzed by Martine M. Mirrione and Daniela Schulz. Experiments were performed and figures prepared by Martine M. Mirrione. All authors contributed to final data analysis and writing.

## Conflict of interest statement

Dr. Henn has received honoria from Wyeth and Bristol Myers Squib for seminar presentations. The other authors declare that the research was conducted in the absence of any commercial or financial relationships that could be construed as a potential conflict of interest.

## Supplementary material

Supplemental Figure 1 **Labeled whole-brain ROI template overlaid on T2-weighted rat template**. Each of 90 coronal sections are shown, where each region is labeled with the designated abbreviation at (or near) the first time it appears in the whole-brain template (abbreviations given below, most abbreviations designated for a prominent sub-region within ROI following (Paxinos and Watson, 2007); Paxinos and Watson, or named as the abbreviation of the general area). Approximate stereotaxic atlas coordinates are displayed for the coronal planes in each row. Each region is color coded so it can be followed on neighboring coronal sections. [Olfactory cortex (Olf.), motor cortex (M1/2), prelimbic cortex (PrL), orbital cortex (O), infralimbic cortex (IL), cingulate cortex (Cg), peduncular cortex (DP), endopiriform cortex (En), insula cortex (I), sensory cortex (S1/2), nucleus accumbens (Acb), caudate (CPu), lateral septum (LS), medial septum (MS), bed nucleus of the stria terminalis, ventral pallidum, and extended amygdala combined area (ST), preoptic area (PO), amygdala cortex (ACo), globus palidus (GP), posterior septum contains triangular septum, septofimbral nucleus and fimbria (TS), hypothalamic nuclei (Hypo), medial thalamic nuclei (mThal.), amygdala nuclei (Amyg.), thalamus (Thal.), retrosplenial cortex (RS), dorsal hippocampus (DHipp.), habenula (Hb), auditory cortex (Au), entorhinal cortex (Ent), parietal cortex (Pt), geniculate (G), zonaincerta (ZI), mammillary nuclei (M), visual cortex (V1/2), ventral hippocampus (VHipp.), substantia nigra (SN), ventral tegmental area (VTA), central gray nuclei (p1, referring to p1PAG area and neighboring nuclei), superior colliculus (Su), red nucleus (RC), interpeduncular nuclei (IP), periquiductal gray (PAG), inferior colliculus (IC), reticular area (Rt), dorsal raphe (DR), rostromedial tegmental nucleus (RMTg), pontine nuclei (Pn), caudal linear raphe (CLi), subiculum transition area (STr), median raphe (MnR), pontine reticular area (PnO), dorsal tegmental area (DTg), cerebellum (Cb), medulla nuceli (Md), cerebellar white matter (cbw), medulla and pons (Md/Pons), cerebellar nuclei (CbN), vermis (CbV)].
