# Supplementary figures and images for: Increased metabolic activity in the septum and habenula during stress is linked to subsequent expression of learned helplessness behavior
Source: Front Hum Neurosci. 2014 Feb 3;8:29. doi: 10.3389/fnhum.2014.00029 (PMC3909949; doi:10.3389/fnhum.2014.00029)

Bregma:

5.16 mm

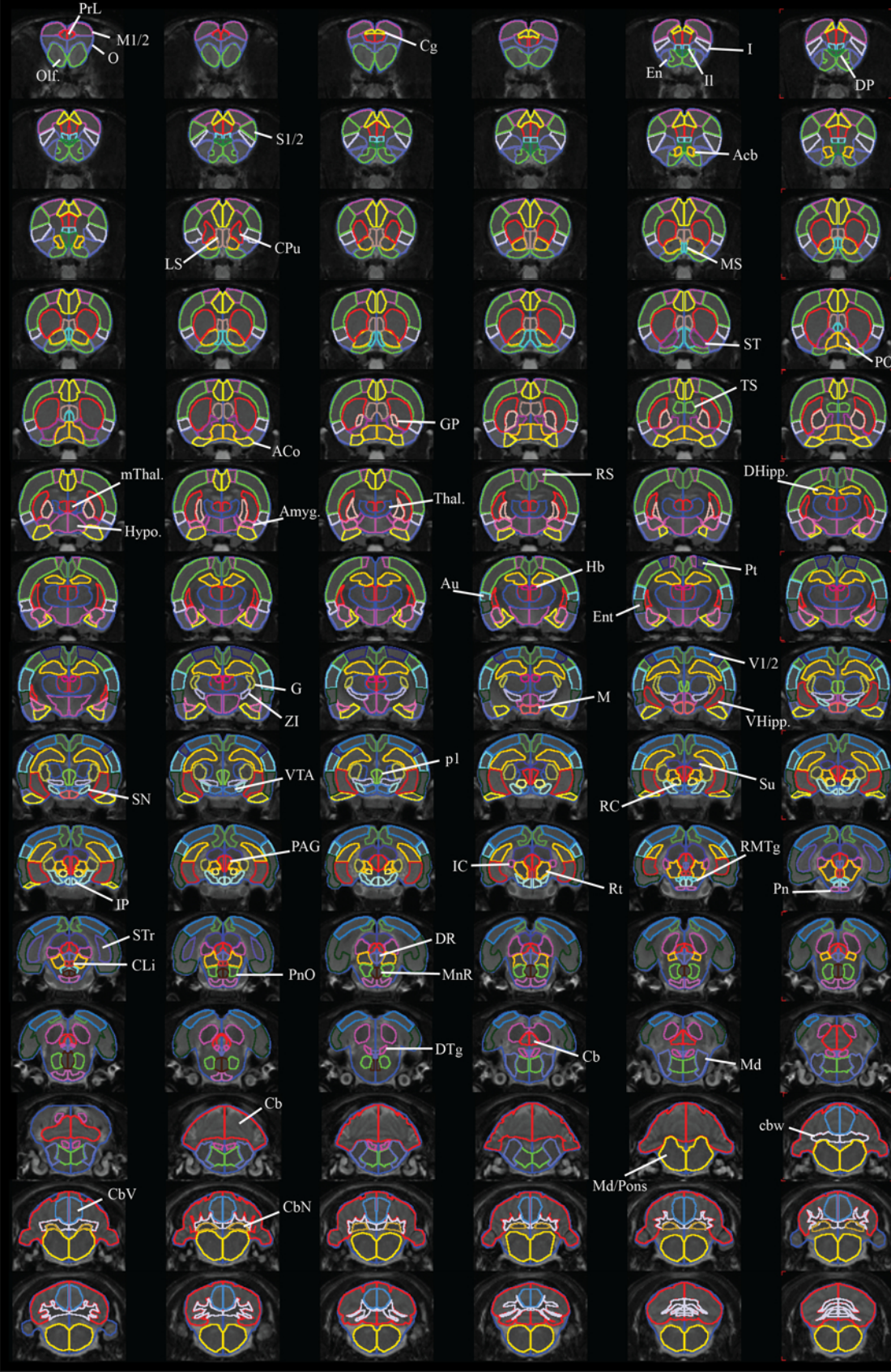

Supplement: Supplemental Figure 1 — Labeled whole-brain ROI template overlaid on T2-weighted rat template. Each of 90 coronal sections are shown, where each region is labeled with the designated abbreviation at (or near) the first time it appears in the whole-brain template (abbreviations given below, most abbreviations designated for a prominent sub-region within ROI following (Paxinos and Watson, 2007); Paxinos and Watson, or named as the abbreviation of the general area). Approximate stereotaxic atlas coordinates are displayed for the coronal planes in each row. Each region is color coded so it can be followed on neighboring coronal sections. [Olfactory cortex (Olf.), motor cortex (M1/2), prelimbic cortex (PrL), orbital cortex (O), infralimbic cortex (IL), cingulate cortex (Cg), peduncular cortex (DP), endopiriform cortex (En), insula cortex (I), sensory cortex (S1/2), nucleus accumbens (Acb), caudate (CPu), lateral septum (LS), medial septum (MS), bed nucleus of the stria terminalis, ventral pallidum, and extended amygdala combined area (ST), preoptic area (PO), amygdala cortex (ACo), globus palidus (GP), posterior septum contains triangular septum, septofimbral nucleus and fimbria (TS), hypothalamic nuclei (Hypo), medial thalamic nuclei (mThal.), amygdala nuclei (Amyg.), thalamus (Thal.), retrosplenial cortex (RS), dorsal hippocampus (DHipp.), habenula (Hb), auditory cortex (Au), entorhinal cortex (Ent), parietal cortex (Pt), geniculate (G), zonaincerta (ZI), mammillary nuclei (M), visual cortex (V1/2), ventral hippocampus (VHipp.), substantia nigra (SN), ventral tegmental area (VTA), central gray nuclei (p1, referring to p1PAG area and neighboring nuclei), superior colliculus (Su), red nucleus (RC), interpeduncular nuclei (IP), periquiductal gray (PAG), inferior colliculus (IC), reticular area (Rt), dorsal raphe (DR), rostromedial tegmental nucleus (RMTg), pontine nuclei (Pn), caudal linear raphe (CLi), subiculum transition area (STr), median raphe (MnR), pontine reticular area (PnO), dorsal teg [file Presentation1.PDF]
